# C1q propagates microglial activation and neurodegeneration in the visual axis following retinal ischemia/reperfusion injury

**DOI:** 10.1186/s13024-016-0089-0

**Published:** 2016-03-24

**Authors:** Sean M. Silverman, Byung-Jin Kim, Garreth R. Howell, Joselyn Miller, Simon W. M. John, Robert J. Wordinger, Abbot F. Clark

**Affiliations:** North Texas Eye Research Institute, University of North Texas Health Science Center, CBH-441, 3500 Camp Bowie Boulevard, Fort Worth, TX 76107 USA; The Jackson Laboratory, Bar Harbor, 04609 ME USA; Alabama State University, Montgomery, AL 36104 USA; Howard Hughes Medical Institute, Bar Harbor, ME 04609 USA

**Keywords:** Retinal ischemia, C1q, Neuroinflammation, Microglia, Astrocytes, Neuroprotection

## Abstract

**Background:**

C1q represents the initiating protein of the classical complement cascade, however recent findings indicate pathway independent roles such as developmental pruning of retinal ganglion cell (RGC) axons. Furthermore, chronic neuroinflammation, including increased expression of C1q and activation of microglia and astrocytes, appears to be a common finding among many neurodegenerative disease models. Here we compare the effects of a retinal ischemia/reperfusion (I/R) injury on glial activation and neurodegeneration in wild type (WT) and *C1qa*-deficient mice in the retina and superior colliculus (SC). Retinal I/R was induced in mice through elevation of intraocular pressure to 120 mmHg for 60 min followed by reperfusion. Glial cell activation and population changes were assessed using immunofluorescence. Neuroprotection was determined using histological measurements of retinal layer thickness, RGC counts, and visual function by flash electroretinography (ERG).

**Results:**

Retinal I/R injury significantly upregulated C1q expression in the retina as early as 72 h and within 7 days in the superficial SC, and was sustained as long as 28 days. Accompanying increased C1q expression was activation of microglia and astrocytes as well as a significantly increased glial population density observed in the retina and SC. Microglial activation and changes in density were completely ablated in *C1qa*-deficient mice, interestingly however there was no effect on astrocytes. Furthermore, loss of *C1qa* significantly rescued I/R-induced loss of RGCs and protected against retinal layer thinning in comparison to WT mice. ERG assessment revealed early preservation of b-wave amplitude deficits from retinal I/R injury due to *C1qa*-deficiency that was lost by day 28.

**Conclusions:**

Our results for the first time demonstrate the spatiotemporal changes in the neuroinflammatory response following retinal I/R injury at both local and distal sites of injury. In addition, we have shown a role for C1q as a primary mediator of microglial activation and pathological damage. This suggests developmental mechanisms of C1q may be re-engaged during injury response, modulation of which may be beneficial for neuroprotection.

**Electronic supplementary material:**

The online version of this article (doi:10.1186/s13024-016-0089-0) contains supplementary material, which is available to authorized users.

## Background

Ischemic events in the CNS cause traumatic tissue damage and irreversible loss of neurons present at the ischemic core and surrounding areas. The metabolic demands of the retina are among the highest of any tissue within the body, receiving a dual blood supply from both the choriocapillaris and the central retinal artery (CRA) [1]. Thus, transient retinal ischemic attacks often cause permanent tissue damage resulting in irreversible vision loss. Following ischemic events in the CNS, there is a significant and detrimental upregulation of biological factors including excess ion influx, excitatory neurotransmitter release, free radical formation, and inflammation [2–4]. Neuroinflammation represents a complex event in the CNS, often involving an activated cellular response from resident immune cells. Once activated, these glial cells secrete a host of pro-inflammatory proteins propagating a chronic cascade of apoptotic or phagocytic events [4].

Two of the primary effector cells during neuroinflammation are the microglia and astrocytes. Both constitute two of the resident immune cells of visual system (the retina, optic nerve, and visual centers of the brain). Astrocytes maintain direct contact with the neurons, blood vessels, and other glial cells, providing metabolic support, modulating synaptic activity, and maintenance of the blood-brain barrier [5, 6]. Microglia represent the resident myeloid cells of the CNS, derived from primitive macrophages of the yolk sac that colonize the neuroepithelium prior to the formation of the blood-brain barrier, separating microglia from circulating macrophages. In addition to their antigen-presenting and phagocytic capabilities, their processes are constantly in a mode of surveillance [5, 7–10]. However, when glia are stimulated by disease or injury, they shift to an activation state leading to the production of inflammatory mediators such as chemokines, cytokines, and complement proteins [11–13]. Activation of brain astrocytes causes a dramatic upregulation by at least four-fold of over 260 genes compared to that of quiescent astrocytes, with some genes showing 10 to 100-fold changes in expression [14]. Accompanying these changes, reactive astrocytes undergo cell proliferation, somatic hypertrophy, overlapping of processes, and scar formation [15]. Likewise, microglia when stimulated undergo a well-defined morphological transformation, proliferation and migration, as well as expression of adhesion molecules [16]. These reactive microglia are often observed at sites of pathology in several neurodegenerative diseases including Alzheimer’s disease and glaucoma [17]. Coupled with their secretion of pro-inflammatory factors such as TNF-α, IL-1β, and C1q, microglia are an ideal candidate as a primary mediator of damage.

C1q, the initiating protein of the classical Complement cascade, is a large complex comprised of six A, six B, and six C chains. Each chain contains a globular region at the carboxyl terminus and a collagen-like stem region at the amino-terminus [18]. Traditionally, the classical pathway is the antibody-dependent activation of complement, as C1q is known to bind to the surface of foreign substances and antibodies [19]. However, recent evidence demonstrates classical complement proteins contribute to various neurodegenerative and age-related diseases [20]. In the DBA/2 J model of spontaneous glaucoma, *C1qa* was among the earliest differentially regulated genes not only in the retina, but also at the optic nerve head (ONH) prior to onset of a glaucomatous phenotype [21, 22]. More surprisingly, during development in the dorsal lateral geniculate nucleus (dLGN), C1q colocalized with either immature pre- or post-synaptic markers, and loss of C1q resulted in retention of overlapping inappropriate connections [22, 23]. Furthermore, C1q expression in certain areas of the brain was found to increase more than 300-fold during normal aging in the mouse. Reduced levels of cognitive and memory decline were observed in aged *C1qa*-knockout mice compared with age-matched WT mice [24].

In this study, we demonstrate the impact of the neuroinflammatory response following an ischemic event in the retina. We identify time dependent increases in cell density and morphological changes of both astrocytes and microglia not only in the retina but also the superior colliculus, a primary termination site of retinal ganglion cell (RGC) axons exiting the retina. Furthermore, we observed significant increases in C1q expression correlating with reactive gliosis. Our goal was to determine if genetic deletion of *C1qa* could morphologically and functionally protect the retina from pathological changes resulting from retinal I/R injury that we have previously characterized [25], as well as attenuate the activation of glial cells in the visual system.

## Results

### C1q expression in the visual axis following I/R injury

**Fig. 1:**
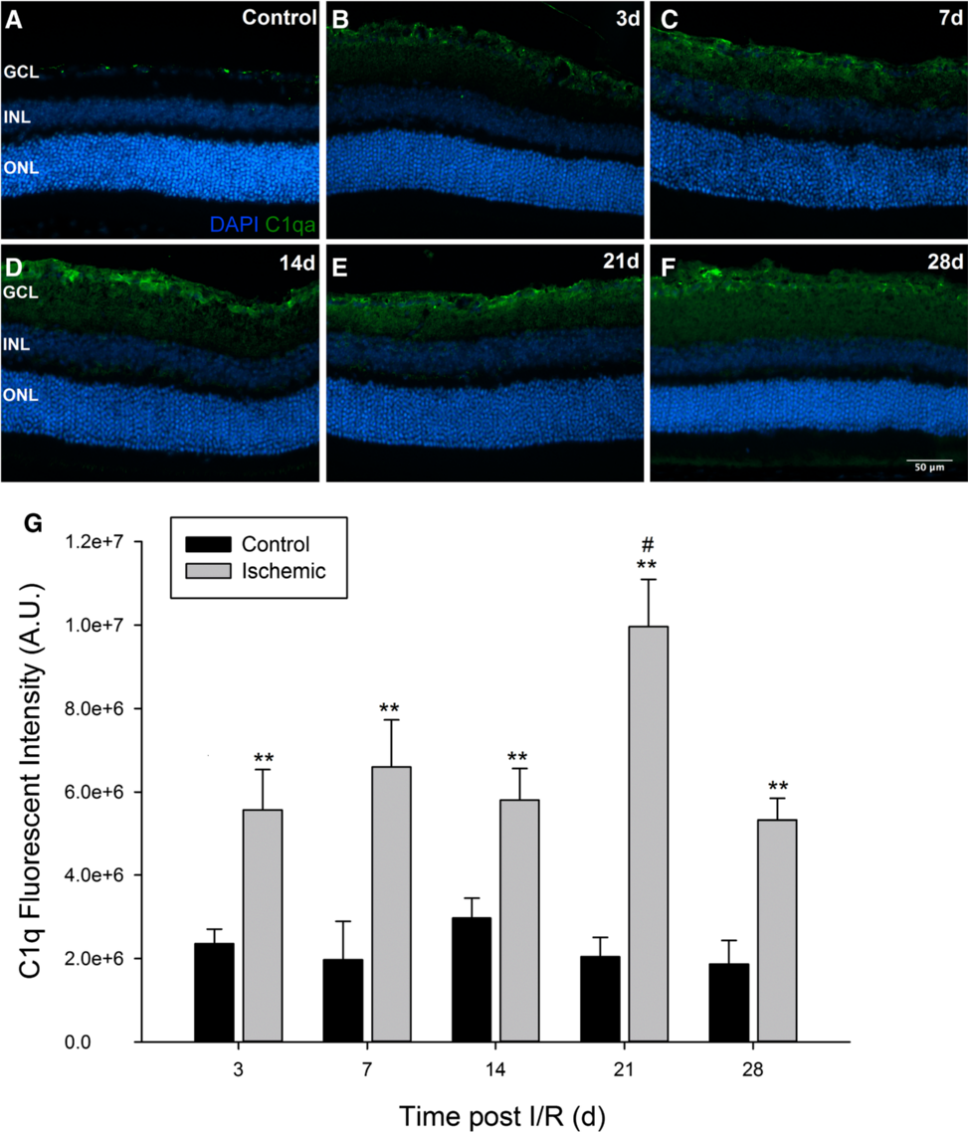
Retinal I/R injury significantly increases C1q expression in the retina. C57BL/6 J mice were subjected to unilateral I/R injury and sacrificed at the indicated time points post I/R. Contralateral control eyes (**a**) displayed basal deposits of C1q in the GCL; however, ischemic eyes (**b**–**f**) had significantly greater expression of C1q as early as day 3 (**b**) and sustained to the end of the time course on day 28 (**f**). Positive labeling was only observed in the GCL and IPL. (**g**) Fluorescence intensity quantification revealed statistically significant upregulation of C1q (*p* < 0.01) at all time points in ischemic versus uninjured retinas. Peak intensity in ischemic eyes was observed on day 21 post injury (*p* < 0.05). Mean ± SEM, *n* = 7 per group. **p < 0.01 determined by student’s paired *t*-test, #*p* < 0.05 tested using One-way ANOVA followed by Holm-Sidak post test. GCL = Ganglion Cell Layer, INL = Inner Nuclear Layer, ONL = Outer Nuclear Layer. Scale bars = 50 μm

Upregulation of complement expression is well documented following ischemic injury; however, temporal changes in C1q expression after retinal I/R have not been identified. In eyes receiving one hour of ischemic injury, C1q was observed primarily in the inner neural retina, extending from the nerve fiber layer (NFL) through the inner plexiform layer (IPL) (Fig. 1a–f). Quantification of fluorescence intensity revealed statistically significant differences (*p* < 0.01) between ischemic (5.5 × 10^6^ ± 9.6 × 10^5^) and uninjured eyes (2.4 × 10^6^ ± 3.5 × 10^5^) as early as day 3, and remaining elevated as long as 28 days (5.3 × 10^6^ ± 5.1 × 10^5^) (Fig. 1g), with a peak intensity 21 days post injury. No changes were observed between uninjured eyes of experimental mice and retinas from naïve mice.

**Fig. 2:**
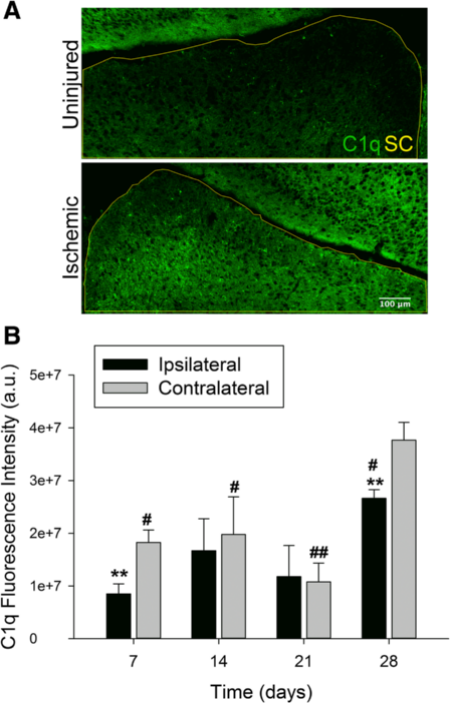
Local ischemic injury in the retina increases expression of C1q in the SC. (**a**) Representative images displaying differences in C1q expression between hemispheres of the SC 7 days following retinal I/R. Yellow outline indicates area of SC measured, scale = 20×. (**b**) Quantification of fluorescence intensity indicated significant differences between ipsilateral (uninjured) and contralateral (ischemic) hemispheres on days 7 and 28 (*p* < 0.01). C1q expression was greatest on day 28 in both hemispheres of the SC, and significantly different versus all time points assessed. Mean ± SEM, *n* = 7 per time point. **p < 0.01 using student’s paired *t*-test between hemispheres within a time point, #*p* < 0.05 ##*p* < 0.01 compared across time points within a group determined via One-way ANOVA followed by Holm-Sidak post test. Scale bars = 100 μm

The primary termination site of a majority of RGC axons in rodents is in the superficial superior colliculus (SC) of the optic tectum. A significant majority of the axons have been shown to cross the optic chiasm and synapse with the hemisphere of the SC contralateral to the eye from which the axons are projecting [26]. Our lab has previously identified the specific areas of the superficial SC innervated by RGC axons using the retrograde fluorescent tracer cholera toxin β (CTB 488) [27]. The RGC axons synapse with relay neurons in order to transfer signals from the retina to the visual cortex. We investigated if a local injury to the retina caused changes in C1q expression at these distal sites in the visual axis. On day 7 the SC hemisphere contralateral to the ischemic eye, henceforth labeled the ischemic hemisphere, displayed a significant elevation in C1q expression in comparison to the ipsilateral hemisphere receiving input from the uninjured retina (Fig. 2a). Basal expression of C1q was also observed in the cortex but appeared to be unaffected by retinal I/R. As in the retina, we quantified differences in C1q expression between the two hemispheres over the 28 day time course. Significant elevation of C1q expression was observed as early as day 7 between hemispheres corresponding to the ischemic (1.8 × 10^7^ ± 2.4 × 10^6^) and uninjured (8.5 × 10^6^ ± 1.9 × 10^6^) eyes. Interestingly by day 14, although not significant, an increase in C1q was apparent in the ipsilateral hemisphere. A second wave of C1q activity was observed at day 28 post injury, displaying peak intensity in both hemispheres. Expression of C1q in the contralateral SC on day 28 was statistically significant (*p* < 0.05) compared to all earlier time points in (Fig. 2b).

### Ischemic induced gliosis in the visual axis

**Fig. 3:**
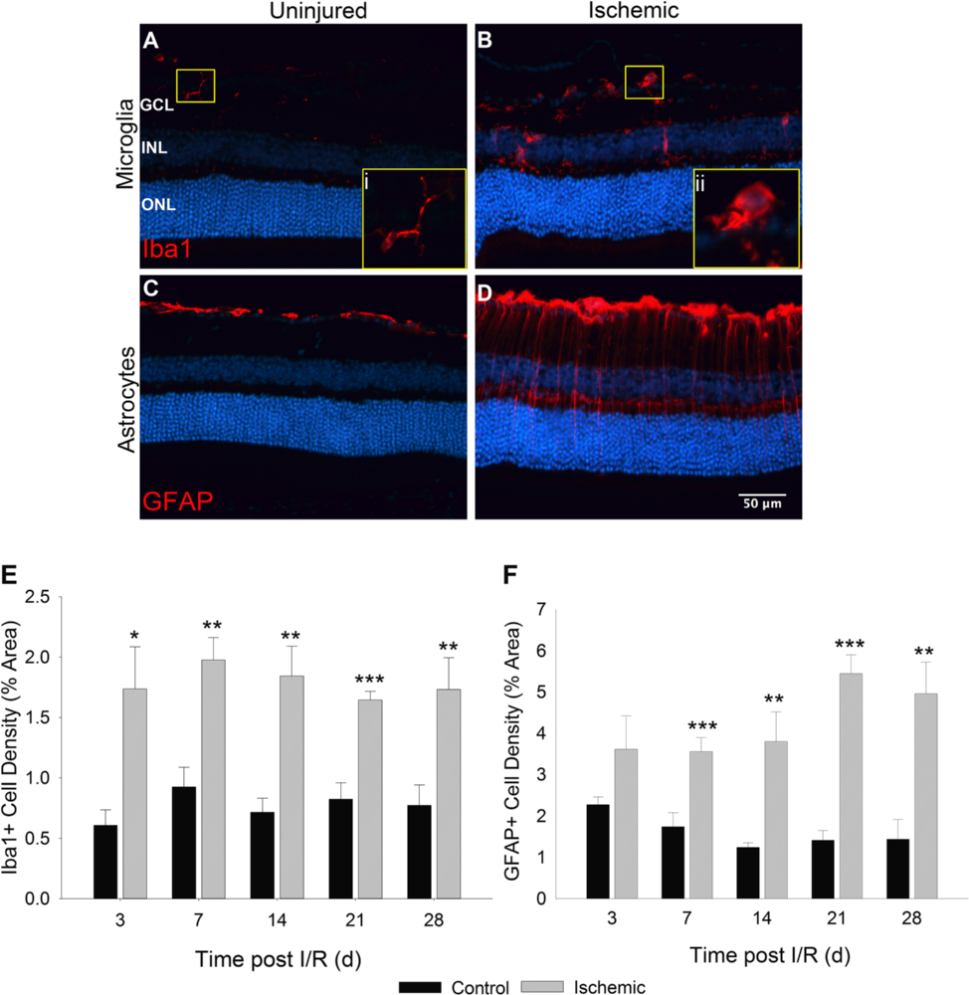
Activation and increased cell populations of macroglia and microglia in the retina post I/R. Representative images of Iba1- and GFAP-positive cells in uninjured (**a**), (**c**) and ischemic (**b**), (**d**) retinas. **a**, **b** Yellow inset boxes of selected Iba1^+^ microglia demonstrate morphological differences between senescent (i) and activated (ii) cellular states. **c**, **d** Morphological differences observed in GFAP^+^ cells following ischemia. Astrocytes and Müller cells display elongated processes extending through the layers of the retina in addition to thickened cell bodies present in the GCL and NFL. **e**, **f** Thresholding analysis based upon percent area of retina occupied was applied to determine changes in cell density. Microglial density (**e**) was significantly increased in ischemic retinas at all time points quantified compared with uninjured controls. Density of macroglia (**f**) trended upward on day 3 and was statistically significant at all time points measured thereafter. Mean ± SEM, *n* = 7 per group. *p < 0.05 **p < 0.01 ***p < 0.001 using students paired *t*-test between control and ischemic groups. GCL = Ganglion Cell Layer, INL = Inner Nuclear Layer, ONL = Outer Nuclear Layer

Ischemic attacks in the CNS are known to activate resident glial cells [4] therefore, we investigated spatiotemporal changes in both microglia and astrocytes (including Müller cells in the retina) accompanying upregulation of C1q following retinal I/R injury. As expected, representative images (Fig. 3a–d) display morphological differences identified between glial cells of uninjured (Fig. 3a and c) and ischemic (Fig. 3b and d) retinas. Activation of microglia is commonly identified by a change from a resting ramified morphology (Fig. 3i) to an amoeboid shape (Fig. 3ii). Activation of astrocytes and Müller cells is observed by upregulation of GFAP expression, as well as outward extension of their processes from the astrocyte somas and Müller cell end feet in the GCL (Fig. 3d). Quantification of these changes based upon percent area revealed significant differences in Iba1^+^ retinal microglia density in ischemic eyes as early as day 3 (1.73 % ± 0.35), and sustained over the entire 28 day time course (*p* < 0.01) compared to contralateral retinas (0.61 % ± 0.13) (Fig. 3e). While GFAP does not distinguish between astrocytes and Müller cells, there was an observed increase in GFAP^+^ cells beginning on day 3; however, a statistically significant difference between ischemic (3.56 % ± 0.34) and uninjured eyes (1.74 % ± 0.34) was not seen until day 7 (Fig. 3f). Increased density of GFAP^+^ cells was significantly different throughout our time course (*p* < 0.01), displaying a gradual increase as pathology progressed. No significant changes were observed in any glial cell type between uninjured contralateral retinas and those of naïve mice.

**Fig. 4:**
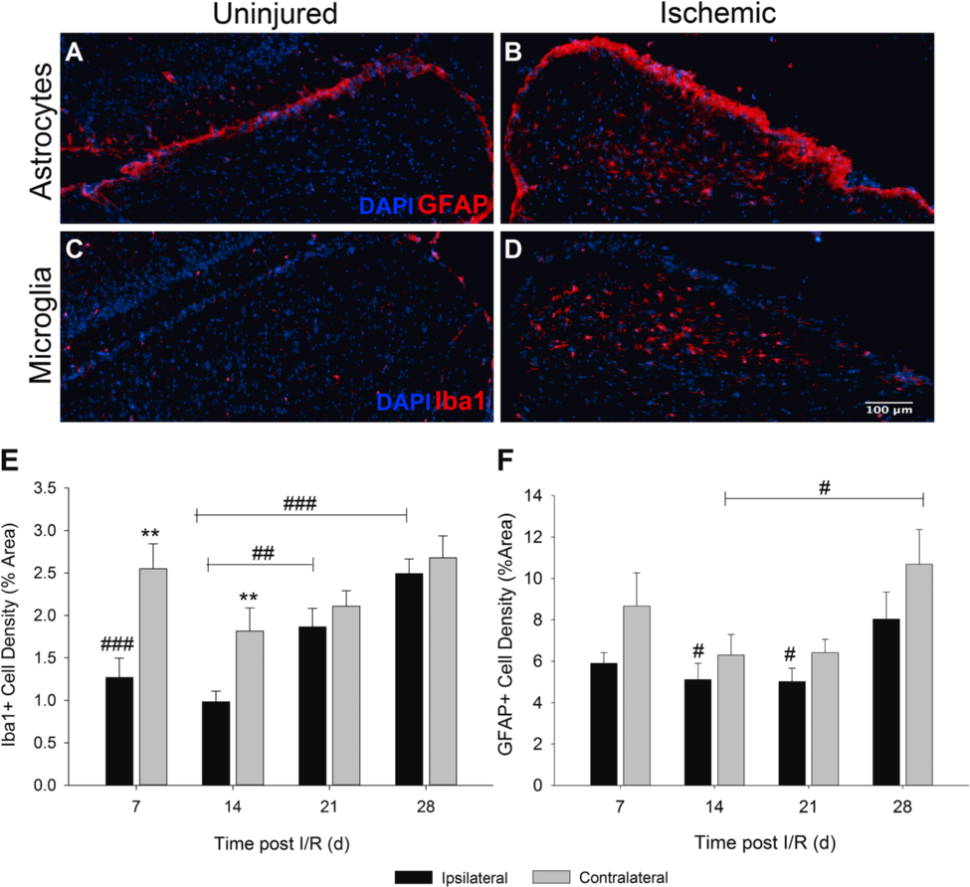
Retinal I/R activates and increases glial cell populations in the SC. Representative images demonstrate differences observed in astrocytes (**a**), (**b**) and microglia (**c**), (**d**) between uninjured ipsilateral and I/R-receiving contralateral hemispheres of the SC. (**e**) Quantification of microglial density was observed to be significantly different between hemispheres on days 7 and 14 (*p* < 0.01) following I/R. No differences were detected between hemispheres by 21 days post injury. However, a statistically significant difference was determined in the uninjured ipsilateral hemisphere when days 21 (*p* < 0.01) and 28 (*p* < 0.001) were compared to earlier time points. (**f**) Density of GFAP^+^ astrocytes was quantified; however, no significant differences could be determined between SC hemispheres at any time point following retinal I/R. However, statistical differences (*p* < 0.05) were observed in both ipsilateral and contralateral hemispheres when day 28 was compared against astrocyte density on days 14 and 21. Mean ± SEM, *n* = 7 per group. **p < 0.01 using students paired *t*-test between ipsilateral and contralateral hemispheres, #*p* < 0.05 ##*p* < 0.01 ###*p* < 0.001 across time points determined by One-way ANOVA followed by Holm-Sidak post test. Scale bars = 100μm

Given that the upregulation of C1q in the retina was accompanied by reactive gliosis, we reasoned a similar effect would be observed in the SC. Similar to our observation of retinal C1q expression, there was a marked difference in glial cell activity in the superficial layers of the SC, correlating to regions of synaptic termination sites of RGC axons (Fig. 4a–d). Both astrocytes (Fig. 4b) and microglia (Fig. 4d) were observed in a reactive morphological state in the contralateral hemispheres from ischemic eyes in comparison to the ipsilateral hemisphere as early as 7 days post injury. Cell density of both Iba1^+^ and GFAP^+^ cells was quantitated in a similar method to our retinal analysis. A very statistically significant (*p* < 0.001) difference in microglial density between hemispheres was seen as early as day 7 (2.56 % ± 0.29 compared to 1.27 % ± 0.23). Density of microglia appeared to drop by day 14, and in a biphasic manner steadily returned on day 28 to peak levels observed on day 7 (Fig. 4e). No significant differences were observed in astrocyte population density between contralateral and ipsilateral hemisphere measurements (Fig. 4f). Interestingly, microglia and astrocyte densities in the hemispheres ipsilateral from injured eyes began significantly increasing on day 21 (*p* < 0.01) for microglia and day 28 (*p* < 0.05) for astrocytes (Fig. 4e, f). Whereas previously significant differences between SC hemispheres could be determined in Iba1^+^ populations, by day 21 both hemispheres were indistinguishable. Our quantification reveals concurrent and progressive increases in C1q expression and glial cell activity in both local and distal tissue sites of the visual axis following retinal I/R.

### C1q mediates morphological damage in the retina

**Fig. 5:**
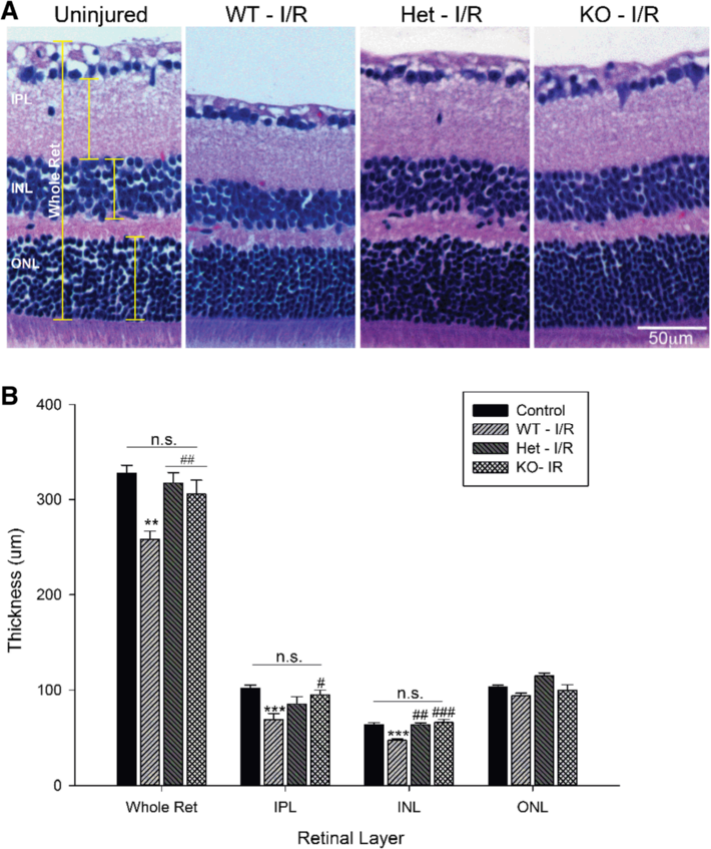
*C1qa*-deficiency protects against retinal thinning resulting from ischemic injury. (**a**) Representative H&E stained retinal cross-section images demonstrating the differences in retinal morphology observed in animals receiving retinal I/R injury compared with an uninjured retina. Retinas from WT mice were observed to have significant thinning of the IPL and INL, as well as dramatic loss of the NFL. However, retinas from *C1qa* ^*+/−*^ and *C1qa* ^*−/−*^ showed no visible changes in thickness. (**b**) Retinal layer thickness analysis of the whole retinal, the inner plexiform layer (IPL), inner nuclear layer (INL), and outer nuclear layer (ONL). A significant difference was determined between control and WT retinas (*p* < 0.01) in all layers measured except for the ONL. This difference was not observed in *C1qa* ^*+/−*^ and *C1qa* ^*−/−*^ retinas. Further, a significant rescue of retinal thickness could be seen between WT, *C1qa* ^*+/−*^, and *C1qa* ^*−/−*^ animals in the whole retina (*p* < 0.01) and the INL (*p* < 0.01), and the IPL (*p* < 0.05) in the *C1qa* ^*−/−*^ mice. Mean ± SEM, *n* = 7 per group. **p < 0.01, ***p < 0.001 compared to control and #*p* < 0.05, ##*p* < 0.01, ###*p* < 0.001 compared to WT–I/R determined by One-way ANOVA followed by Holm-Sidak post test. Scale bars = 50μm

Previously we have published morphological degeneration and altered retinal gene expression resulting from retinal I/R [25]; here we employ several of those same assays to observe these changes in WT, heterozygous, or homozygous mice null for *C1qa*. Retinal I/R caused a thinning of the inner layers of the neural retina associated with a loss in the neurons residing in the GCL, IPL, and INL, the same layers in which we observed a prolonged neuroinflammatory response. However, we observed a significant protection of all of these layers in *C1qa*^*+/−*^ and *C1qa*^*−/−*^ mice (Fig. 5a). We further quantified these findings through caliper measurements using ImageJ. Overall, we found no statistical differences between the total thicknesses of uninjured retinas (327.6 ± 7.9 μm) compared with the ischemic retinas of *C1qa*^*+/−*^ (317.0 ± 10.9 μm) and *C1qa*^*−/−*^ (309.5 ± 14.5 μm). Furthermore, a significant difference was quantified in both the IPL (*p* < 0.05) and INL (*p* < 0.001) when comparing WT and *C1qa*^−/−^ mice (Fig. 5b). Control measurements were performed in both naïve and uninjured eyes in both *C1qa* mutant genotypes for phenotypic variation, and no significant differences in individual layer or total retinal thickness were observed (Additional file 1: Figure S1A). All measurements were performed under masked conditions.

**Fig. 6:**
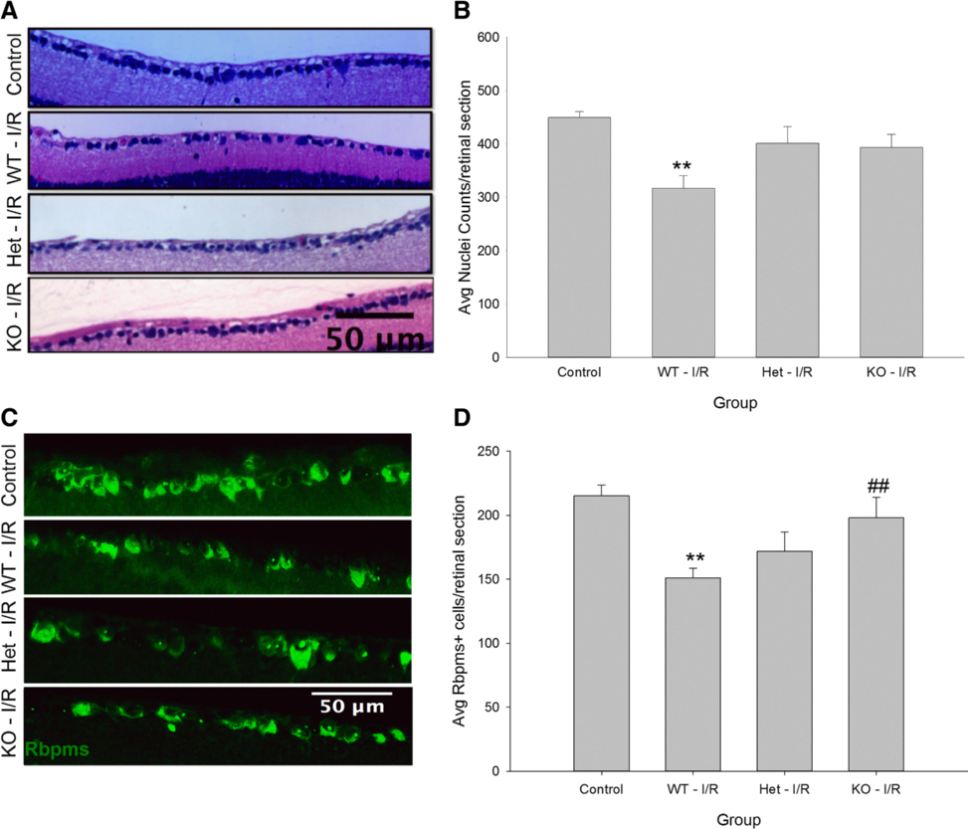
*C1qa*-deficiency mitigates loss of RGCs using two independent assays of cell counts 28 days following retinal I/R. (**a**) H&E stained and (**c**) Rbpms-immunolabeled retinal cross sections used to assess RGC loss between uninjured controls and three *C1qa* genetic backgrounds receiving ischemic injury. (**b**) Quantification of nuclei counted in the GCL determined there was a significant loss of cells (*p* < 0.01) in WT retinas but not in the *C1qa*-deficient retinas compared to control. (**d**) Counts of Rbpms^+^ RGCs displayed a statistically significant loss (*p* < 0.01) in ischemic eyes of WT mice compared to controls. No statistical differences were determined in ischemic retinas of *C1qa* ^*+/−*^ or *C1qa* ^*−/−*^ animals. However, a statistical difference (*p* < 0.01) was measured when *C1qa* ^*−/−*^ retinas were compared to WT. Mean ± SEM, *n* = 7 per group. **p < 0.01 compared to control and ##*p* < 0.01 compared to WT – I/R determined by One-way ANOVA followed by Holm-Sidak post test. Scale bars = 50 μm

In addition to deficits in retinal layer thickness, a significant loss of RGCs occurred following I/R injury. Two independent methods of cell counting were utilized to assess loss of RGCs between ischemic eyes of our three genotypes. Representative images demonstrate the differences observed in the GCL of H&E stained retinas between control and ischemic retinas of WT and *C1qa*-deficient animals (Fig. 6a). Cell counts revealed a statistically significant loss (*p* < 0.01) in ischemic eyes of WT mice (317 ± 23 cells per mm) in comparison to their contralateral uninjured retinas (450 ± 11 cells per mm). However, no statistical differences were determined between control retinas and those from *C1qa*^*+/−*^ and *C1qa*^*−/−*^ mice (401 ± 31 and 394 ± 24 cells per mm, respectively) (Fig. 6b). Since H&E staining is unable to differentiate between RGCs and displaced amacrine cells, the RGC-specific marker RNA-binding protein with multiple splicing (Rbpms) was used for a more accurate characterization (Fig. 6c). Again, there was a significant difference (*p* < 0.01) identified between control (215 ± 8 cell per mm) and ischemic retinas (151 ± 8 cells per mm) of WT mice. This difference was ablated in *C1qa*^+/−^ ischemic eyes (172 ± 15 cell per mm), and further a significant difference was seen when ischemic eyes of WT mice were compared with those of *C1qa*^*−/−*^ animals (198 ± 16 cells per mm, *p* < 0.01) compared to WT (Fig. 6d). There were no differences in RGC counts in contralateral uninjured and naïve retinas of WT and *C1qa*-deficient strains (Additional file 1: Figure S1B and C). All counts were performed in a masked manner.

### Retinal functional deficits are delayed in absence of C1qa

**Fig. 7:**
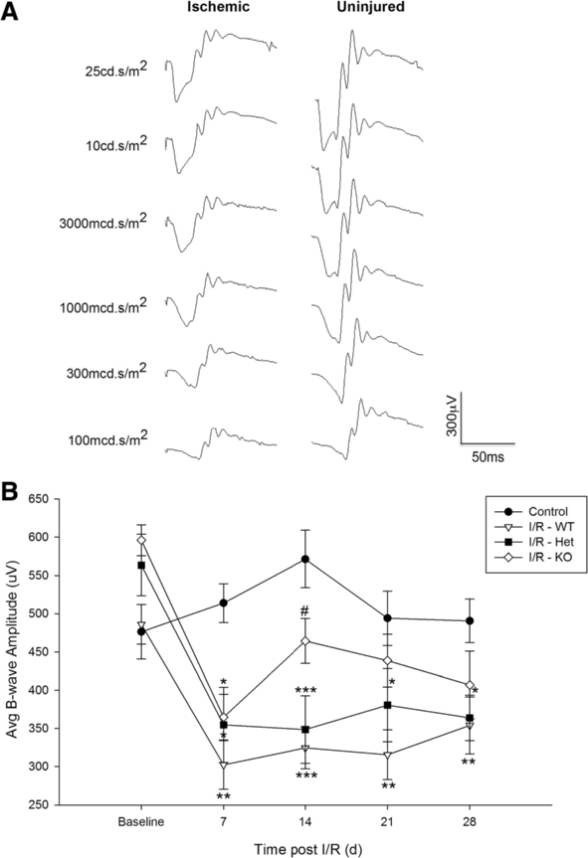
*C1q*-deficiency delays loss of visual function due to retinal I/R. (**a**) Representative ERG traces observed in WT mice comparing waveforms from ischemic and uninjured eyes across a series of flash intensities tested 28 days post injury. (**b**) Comparison of b-wave amplitudes over a 28 day time course at an intensity of 3000 mcd.s/m^2^. A statistically significant loss of b-wave response was observed in WT (*p* < 0.01) and *C1qa* ^+/-^ (*p* < 0.05) ischemic eyes compared to uninjured controls at all time points. However, following initial b-wave suppression, *C1qa* ^-/-^ ischemic eyes displayed a significant rescue (*p* < 0.05) on day 14. This rescue was transient as a slight decline was observed over the remaining two time points; by the end no statistical differences were observed between any animals receiving ischemic injury. Mean ± SEM, *n* = 8 per group. *p < 0.05 **p < 0.01 ***p < 0.001 compared to control #*p* < 0.05 comared to WT–I/R determined by One-way ANOVA followed by Holm-Sidak post test

Along with our characterization of morphological deficits, we also used scotopic flash ERG to assess changes in retinal function in our three genetic backgrounds. ERG waveforms can be subdivided into multiple components including the a-wave, or photoreceptor response, and the b-wave, comprising the response from bipolar and Müller cells. The b-wave is measured from the trough of the wave to its peak. Due to changes only observed in the inner retina resulting from retinal I/R, we quantified b-wave amplitudes. Representative traces display differences in ERG b-wave amplitudes between ischemic and control eyes in WT mice (Fig. 7a). An intensity of 3000mcd.s/m^2^ was chosen for quantification. We observed a significant loss in b-wave amplitudes in all animals 7 days following retinal I/R (*p* < 0.05-0.01). While b-wave measurements remained statistically different in WT and *C1qa*^*+/−*^ mice (*p* < 0.01) throughout our study compared to uninjured eyes, a functional rescue was seen in our complete knockouts. *C1qa*^*−/−*^ mice were observed not only to have significantly greater (*p* < 0.05) b-wave readings at day 14 compared to WT (464.3 ± 29.3 μV and 324.5 ± 27.3 μV, respectively), but also there was no statistical difference in comparison to control eyes. This rescue appeared to be temporary, and by the end of our time course on day 28, no discernable differences were observed in any animals receiving I/R injury (Fig. 7b).

### C1q-deficiency modulates glial response in the visual axis after injury

Our studies conducted using WT mice established the extent of reactive gliosis observed through the visual axis over a 28 day time course following retinal I/R injury (Figs. 3 and 4). Given our findings of protection observed in the retina, we hypothesized C1q may be responsible for the activation and density increases observed in glial cells. The same methodology was used to perform density analysis in both the retina and SC in *C1qa*-deficient mice. Initially, significant differences were observed as early as day 7 post injury, and maximum density was measured on day 28. Therefore, we chose these two key time points for our assessment of glial density in the absence of C1q.

**Fig. 8:**
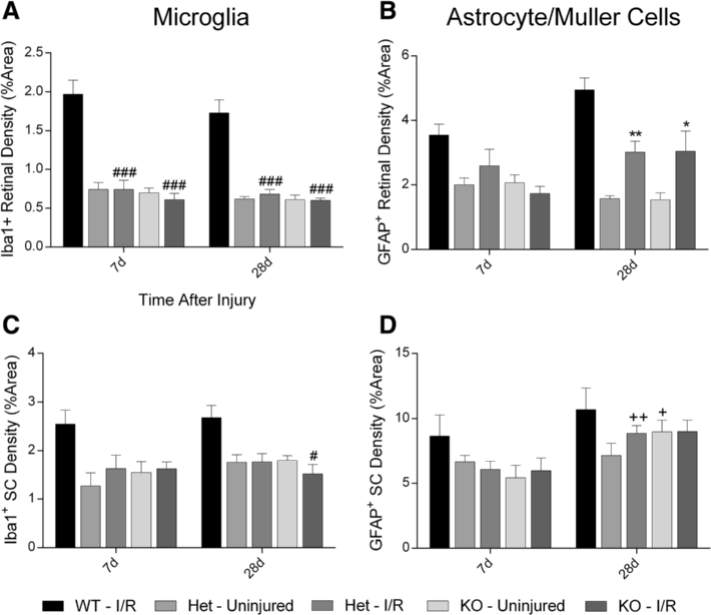
*C1qa*-deficiency modulates the reactive microglial but not astrocyte response in the retina and SC. **a**, **b** Bar graphs display density of Iba1^+^ microglia (**a**) or GFAP^+^ (**b**) cells in the retina at both 7 and 28 days post injury. No statistical changes between uninjured or injured *C1qa* ^*+/−*^ or *C1qa* ^*−/−*^ mice were observed in microglia density at any time point measured; however, there was a statistically significant decrease compared with WT retinal microglia (*p* < 0.001). On day 28 there was significant difference in GFAP^+^ cell density (*p* < 0.05) between ischemic and uninjured retinas of *C1qa* ^*+/−*^ and *C1qa* ^*−/−*^ mice. **c**, **d** Bar graphs demonstrating glial cell density in the SC of *C1qa*-deficient mice. (**c**) Microglial density was unchanged in both the ischemic-receiving and uninjured ipsilateral hemisphere at both 7 and 28 days, and *C1qa* ^*−/−*^ displayed significant decrease compared to peak microglial density at day 28 (p < 0.05). (**d**) No differences were determined between hemispheres on day 7 or 28 in astrocyte density, but there was a significant increase in density on day 28 when compared to day 7 in *C1qa* ^*+/−*^ (*p* < 0.01) and *C1qa* ^*−/−*^ (*p* < 0.05) mice. No statistical differences were measured when *C1qa*-deficicient were compared to peak WT astrogliosis density. Mean ± SEM, *n* = 6 per group. *p < 0.05 **p < 0.01 compared to uninjured control determined by student’s paired *t*-test. +*p* < 0.05 ++*p* < 0.01 compared across time points within a group determined by student’s *t*-test. #*p* < 0.05, ###*p* < 0.001 compared to glial cell density of I/R injured retinas of WT mice, determined by One-way ANOVA followed by Holm-Sidak post test

In the retina, unlike the changes observed in WT animals, no statistical differences in microglial density were observed between ischemic and contralateral uninjured retinas in either *C1qa*^*+/−*^ or *C1qa*^*−/−*^ mice at 7 and 28 days post injury (Fig. 8a). In addition, injured retinas from *C1qa*-deficient mice displayed significantly decreased (*p* < 0.001) microglial density when compared with WT injured retinas at early and late time points. Likewise for SC microglia, no differences were observed between hemispheres at day 7 and no significant increases in density were measured between days 7 and 28 in either of our *C1qa*-deficient mice, as was previously seen in WT mice (Fig. 8c). However, a different trend was observed in astrocyte activity and density. Although no differences were observed on day 7 in both the retina and SC of *C1qa*-deficient animals, by the end of our time course, significant changes in density were determined. Retinas of *C1qa*^*+/−*^ and *C1qa*^*−/−*^ mice receiving I/R injury displayed statistically significant (*p* < 0.05-0.01) increases in GFAP^+^ cell density (3.02 ± 0.34 and 3.05 ± 0.62 %, respectively) compared to uninjured retinas (Fig. 8b). Similar to our results observed in the SC of WT mice, no difference could be determined between ipsilateral and contralateral hemispheres by day 28, and there was a statistically significant increase in astrocyte density compared to day 7 (*p* < 0.05- 0.01, Fig. 8d). In both the retina and the SC no differences were determined when astrocyte density in *C1qa*^*+/−*^ mice were compared to *C1qa*^*−/−*^ mice. Although the density of astrocytes was reduced when astrogliosis of *C1qa*-deficient mice was compared to WT, no statistical differences were observed. Taken all together, our data indicate reactive gliosis was observed in both astrocytes and microglia in the visual axis of WT animals, but *C1qa-*deficient mice had an ablated microglial response. However, while significant changes were observed in astrocyte density following injury, this response also was dampened prior to onset of pathology in comparison to that measured in WT mice.

## Discussion

Our studies have demonstrated a significant temporal increase in C1q expression that is accompanied by an increase in reactive microglial and astrocytic retinal cell density following retinal I/R injury. Further, this response is mirrored at RGC termination sites in the visual system through increased C1q expression observed in the SC. These findings agree with previous reports indicating the involvement of both the complement system and glial response to ischemic events not only in the retina, but also areas of the brain affected by I/R Injury [4, 12, 28, 29]. The absence of C1q provided retinal neuroprotection through rescue against I/R-induced retinal thinning and neuronal loss. It should be noted that while *C1qa*^*+/−*^ mice do have measureable levels of C1q, following ischemic injury, these levels were significantly reduced in comparison to WT retina and SC (data not shown). Though present, this dramatic reduction likely explains the partially protective phenotype observed in these mice. Additionally, attenuation of the activated microglial response in the retina and SC was observed in *C1qa*-deficient animals. To our knowledge, this is the first report to identify a time-dependent neuroinflammatory response in the visual system following retinal ischemia, and to establish C1q as a primary mediator of damage.

Given the wide variety of animal models of ischemic injury (e.g. global, cardiac, cerebral, or retinal), several methods for neuroprotection have met with mixed results [30]. The retina provides a unique target for testing of neuroprotective compounds as investigators have the option of local [31–33] or systemic [34–36] delivery of therapeutic compounds or biologics. We have demonstrated significant protection against both functional and morphological deficits in the retina and SC subjected to I/R injury using the JNK inhibitor SP600125 (Kim et al., submitted for publication). Time dependent gene profiling following retinal ischemia identifies several members of the complement family to be among the most significant changes as early as 7 days post injury [3, 37]. Previous studies identifying protection of RGCs in a *C3*-deficient mouse following retinal I/R demonstrated a delayed loss of cells after 3 weeks; however, no difference in axonal damage was seen compared with WT mice [3]. While our endpoints differed from Kuehn et al., we were able to show a significant rescue of RGCs and cells in the GCL. While there exists sufficient evidence of activation of the downstream complement cascade including C3 following retinal ischemia, our results suggest C1q may be acting in a cascade-independent role.

Activation of the complement system in the CNS during times of injury and pathogenesis has been well documented [38, 39]. Increased expression of C1q has been specifically observed to colocalize with hallmark pathological changes observed in several neurodegenerative diseases including Alzheimer’s disease (AD), Parkinson’s disease (PD), and glaucoma [20, 40]. Furthermore, C1q is known to directly bind to the membrane of neurons, which have a poor capacity to regulate activation of downstream complement cascade factors [41]. Our studies demonstrate significant upregulation of C1q at both local and distal sites in the visual system following I/R injury. As such, C1q has been identified as a potential prime therapeutic target. Although not indicated for treatment of chronic neurodegenerative disease, previous strategies to modulate C1q have targeted C1r/C1s, which is necessary with C1q to form the C1 complex, using recombinant C1-INH as well as soluble CR1 [42]. In animal models of brain ischemia, administration of C1-INH was observed to reduce infarct volume and neurological deficits [28, 43]. Similar to our findings of cell rescue and retinal morphology preservation, deletion of C1q has been identified as a protective mechanism in various other disease models. Recent in vitro data suggested C1q promoted neurite progression by modulating expression of genes necessary for outgrowth [44]. These findings were later supported, and extended in vivo*,* demonstrating axon regeneration and improved guidance following spinal cord injury [45]. In both studies however, downstream C3 and C5 had non-growth promoting effects, suggesting distinct mechanistic roles for different complement components. AD mice deficient for *C1q* (APPQ^−/−^) significantly preserved functional neurons and increased dendritic staining in the hippocampus compared to Aβ pathological (APP) mice [46]. Accumulation of C1q during normal brain aging has been implicated in cognitive decline; age-matched *C1qa*-deficient mice were observed to perform significantly better in a series of learning and memory behavioral tests compared to WT mice [24]. Further, in the DBA/2 J spontaneous glaucoma model, *C1qa* was identified to be differentially expressed in the retina and ONH preceding phenotypic glaucomatous damage. Deletion of *C1qa* significantly delayed RGC axonal damage [21, 22].

In our current study, we identified for the first time a temporal response pattern for microglia and astrocyte activation in the retina and SC after retinal I/R injury. Furthermore, we demonstrated the significant increase in microglial activation and cell density resulted from accumulation of C1q in the injured tissue. In the CNS, astrocytes, microglia, and neurons have been identified as the primary producers of complement proteins [40, 47]. Our data support previous reports that disturbances to the retina from injury and inflammation results in a dynamic response of microglia, known to phagocytose dying neurons [48–50]. Evidence suggests the increased cell density may result from infiltrating microglia through compromised blood-retinal barriers [51]; however, our methodology was unable to differentiate resident and migratory microglia. Further studies utilizing fluorescently-labeled transplanted cells will be needed to identify whether our observed changes were due to infiltration, proliferation, or a combination of both. Given the implication of chronic reactive microglia in neurodegenerative disease as well as recently discovered developmental roles [52, 53], several approaches have been made to abate microglial activation. Neuroprotective treatment strategies such as minocycline, irradiation, and TSPO have shown significant suppression of microglial activation and proliferation, while other methods such as modulation of fractalkine receptor CX3CR1 have led to mixed results [29, 48, 54–57]. These studies, along with our current work, demonstrate the therapeutic potential of targeting microglia in several animal models of neurodegeneration. Furthermore, early clinical trials using oral minocycline resulted in moderately improved visual acuity and reduction in vascular permeability in patients with diabetic macular edema [58].

Despite our *C1q*-dependent modulation of activated microglia and observed morphological protection in the retina, we were unable to totally rescue I/R induced loss of retinal function as assessed through scotopic ERG. Coinciding with this finding, mice deficient in *C1qa* displayed increased levels of GFAP similar to WT animals following retinal I/R. This supports previous results that microglia are the primary synthesizers of complement proteins, specifically C1q, leading to pathogenesis from prolonged inflammatory responses [7, 56]. This suggests following ischemic episodes in the retina, prolonged astrocyte and Müller cell activation is stimulated by proinflammatory factors different from microglia. Reactive astrogliosis is known to be triggered by cytokines such as TNFα, CNTF, IL-1β, and IL-6 [59]. Further, these macroglial cells facilitate a certain degree of neurodegeneration independent from microglia, which may explain our findings of visual deficits, as well as the second wave of C1q expression observed in the SC on day 28. Similar to microglia, astrocytes posses the machinery for synaptic engulfment; however, this appears to be a C1q-independent process [60]. GFAP represents an essential intermediate filament of reactive astrocytes necessary for the many astroglial functions following injury [61]. Genetic deletion of GFAP has provided mixed results of protection and exacerbation that appear to be dependent on the nature of the injury [62–64]. Astrocytes, both neighboring and tissue-specific, have been identified as a highly heterogenous population in their gene expression patterns, morphology, and proliferative responses to injury, which are further complicated by their proximity to the trauma [15]. Therefore, it is likely that retinal astrocytes would react much differently to retinal I/R injury compared to astrocytes in the SC. Therefore, we find it unsurprising that elimination of *C1q* had no significant effect on controlling this gliotic response in the SC. Combinatorial therapies have had recent success in improving pathological endpoints in models of injury and disease [65]; therefore, targeting multiple proinflammatory proteins may further suppress the glial response to I/R injury, thereby providing enhanced neuroprotection.

## Conclusions

In summary, we have identified the temporal expression patterns of C1q and the subsequent glial response to retinal I/R injury to neurons in both the retina and SC. Further, we demonstrate that retinal I/R induced neurodegeneration requires C1q expression, and that genetic ablation of C1q completely inhibited the reactive microglial response leading to preservation of retinal morphology and significant protection of RGCs compared with WT mice. However, retinal astrocyte and Müller cell activation persisted likely causing the transient functional retinal protection observed. Our results are the first to identify a relationship between C1q and microglia following retinal injury, which we hypothesize to be directly related to their roles in synaptic maturation in the developing visual system [53]. Greater understanding of the similarities between developmental and neurodegenerative signaling represents exciting new avenues for therapeutic intervention and enhanced neuroprotection of the visual axis.

## Methods

### Animals

Male and female transgenic mice for *C1qa* (B6.129P2-C1qa < tmlmjw>/Sj) that were backcrossed 10 generations onto a pure C57BL/6 J background and (hereafter referred to as either *C1qa*^*+/−*^ or *C1qa*^*−/−*^*)*, were generously provided from the Simon John laboratory (Jackson Laboratory; Bar Harbor, ME). Female C57BL/6 J (Jackson Laboratory) and male and female *C1qa* mice (3–4 months of age) were used for transient retinal I/R studies. Animals were maintained in 12-h:12-h/light:dark cycle. All studies and animal care were performed as approved by the Institutional Animal Care and Use Committee at the University of North Texas Health Science Center and followed the Association for Research in Vision and Ophthalmology (ARVO) Statement for the Use of Animals in Ophthalmic and Vision Research.

### Retinal I/R

Retinal I/R was induced as described previously [25]. Briefly, mice were anesthetized with a ketamine/xylazine/acepromazine cocktail (100/10/3 mg/kg), and the left eyes were dilated (2.5 % Phenylephrine HCl; Paragon BioTeck, Inc.) followed by cannulation of the anterior chamber with a 30-gauge needle connected to a reservoir containing sterile PBS. The reservoir was elevated to generate an intraocular pressure of 120 mmHg for 1 h to induce retinal ischemia. Afterwards, the cannula was removed and blood was allowed to naturally reperfuse the retina. Body temperature was maintained on a digitally controlled heating pad for the duration of the procedure and recovery.

### Immunofluorescence of the retina and SC

Following euthanasia of the mice, ischemic and contralateral control eyes were gently removed and placed in 4 % paraformaldehyde (PFA) for 4 h followed by a 20 % sucrose solution overnight. Whole brains were delicately excised and fixed in 4 % PFA for 6–8 h, after which a 3 mm region was cut to include the superior colliculus (SC) using a mouse brain block. The SC-containing brain section was also embedded in a 20 % sucrose solution overnight. Eyes and brains were placed in Tissue-Tek OCT compound (Sakura Finetek USA, Torrance, CA) and frozen over dry ice before being stored at −80 °C. Eyes and brains were frozen sectioned at 12 μm and placed on Superfrost Plus slides (VWR, Radnor PA). Slides were triple washed in PBS, blocked and permeabilized for 1 h in blocking buffer (PBS with 10 % fetal bovine serum and 0.15 % Triton X-100) before incubation with primary antibodies overnight at 4 °C. For retinal sections anti-C1q (A201, 1:1000) from Quidel (San Diego, CA) was used; however excessive non-specific labeling was observed with this antibody in SC sections, therefore anti-C1q (ab182451, 1:200) from Abcam (Cambridge, MA) developed by Stephan and colleagues was used [24]. For glial cell analysis, antibodies to Iba1 (019-19741, 1:500) from Wako Chemicals (Richmond, VA) and GFAP (ab7260, 1:300) from Abcam were used. RGC counts were performed using the specific marker Rbpms (GTX118619; 1:200) from GeneTex (Irvine, CA). Tissues were incubated in secondary antibodies conjugated with Alexafluor 488 and Alexaflour 592 (1:250; Invitrogen/Molecular Probes, Carlsbad, CA) for 1 h at room temperature. Slides were mounted with cover slips using ProlongGold anti-fade reagent with DAPI (Molecular Probes, Life Technologies, Grand Island, NY). Images were taken using a Nikon Eclipse Ti inverted microscope (Nikon; Melville, NY) and CRi Nuance FX multispectral imaging system (Caliper Life Sciences; Hopkinton, MA). Autofluorescence was subtracted using Nuance 3.0 software (Caliper Life Sciences).

### Cell density analysis

Density of astrocytes, Müller cells and microglia in the retina and SC was calculated using thresholding analysis in ImageJ (NIH; Bethesda, MD). Briefly, images were separated by color channel into an RGB stack, after which a region of interest (ROI) was drawn around the entire tissue. A threshold was set to positively select only Iba1^+^ or GFAP^+^ cells, and this threshold was maintained for all images analyzed. Measurements were limited to this threshold and the percent area occupied of the section was determined. A minimum of three images per section and three slides spanning the depth of the tissue were analyzed and averaged per animal. A similar methodology was used to assess fluorescence intensity of C1q in retina and SC sections.

### Histological assessment of the retina

Whole globes were immersion fixed in 4 % PFA overnight at 4 °C, followed by paraffin processing. Eyes were sectioned at 5 μm and stained with hematoxylin and eosin (H&E). Entire retinas were imaged, ora serrata to ora serrata through the optic nerve head, and thickness was measured using calibrated calipers in ImageJ from the nerve fiber layer (NFL) through the outer nuclear layer (ONL) at two peripheral and two central locations of the retina. Three slides were selected per retina and the four cross sectional measurements from each retina were averaged together. Nuclei in the H&E ganglion cell layer (GCL), including RGCs and displaced amacrine cells, were counted from each retina and averaged.

### Scotopic flash electroretinography (ERG)

All animals were dark adapted overnight; mice were anesthetized with isoflurane and connected to the HMsERG system (Ocuscience; Rolla, MO). Body temperature was maintained at 37 °C. A ground electrode was placed subcutaneously by the tail and reference electrodes inserted under each eye. Silver-thread electrodes were placed across the apex of the cornea and held in place with a Gonak (Akorn; Lake Forest, IL) coated contact lens. Eyes were exposed to a series of light flashes at increasing intensities (0.1, 0.3, 1, 3, 10, and 25 cd.s/m^2^). Amplitudes and implicit times of waveforms were measured and analyzed.

### Statistics

Statistical analysis was performed using SigmaPlot 12 (Systat; San Jose, CA). Student’s paired *t*-test was used to compare experimental groups within animals, ischemic versus contralateral control. One-way ANOVA was used to compare among three or more groups, such as comparing between time points for experimental groups. Holm-Sidak *post hoc* analysis was used for multiple comparisons. All data are expressed as mean ± standard error mean (SEM), and p-values less 0.05 were considered statistically significant.

## Additional file

Additional file 1: Figure S1.
*C1qa*-deficiency does not affect retinal morphology. Naïve retinas from *C1qa+/−* and *C1qa−/−* were compared to those of WT mice in all neuroprotective assays. (A) No statistical differences were observed between all three genotypes in total retinal thickness. Likewise, when total nuclei in the GCL (B) and Rbpms + (C) cells in the retina were counted, no differences were determined. Therefore, quantifications indicating preservation of retinal thickness and neuron counts are not due to developmental differences within the retina between transgenic and WT mice. Mean ± SEM, *n* = 5 per group. (PDF 140 kb)

## Abbreviations

CRA: central retinal artery
dLGN: dorsal lateral geniculate
ERG: electroretinogram
GCL: ganglion cell layer
GFAP: glial fibrillary acidic protein
I/R: ischemia/reperfusion
Iba1: Ionized calcium-binding adapter molecule 1
IPL: inner plexiform layer
NFL: nerve fiber layer
ONH: optic nerve head
RGC: retinal ganglion cell
Rpbms: RNA-binding protein with multiple splicing
SC: superior colliculus
WT: wild type
