# Supplementary material for: C1q propagates microglial activation and neurodegeneration in the visual axis following retinal ischemia/reperfusion injury
Source: Mol Neurodegener. 2016 Mar 24;11:24. doi: 10.1186/s13024-016-0089-0 (PMC4806521; doi:10.1186/s13024-016-0089-0)
Supplement: Additional file 1: Figure S1. — C1qa-deficiency does not affect retinal morphology. Naïve retinas from C1qa+/− and C1qa−/− were compared to those of WT mice in all neuroprotective assays. (A) No statistical differences were observed between all three genotypes in total retinal thickness. Likewise, when total nuclei in the GCL (B) and Rbpms + (C) cells in the retina were counted, no differences were determined. Therefore, quantifications indicating preservation of retinal thickness and neuron counts are not due to developmental differences within the retina between transgenic and WT mice. Mean ± SEM, n = 5 per group. (PDF 140 kb) [file 13024_2016_89_MOESM1_ESM.pdf]

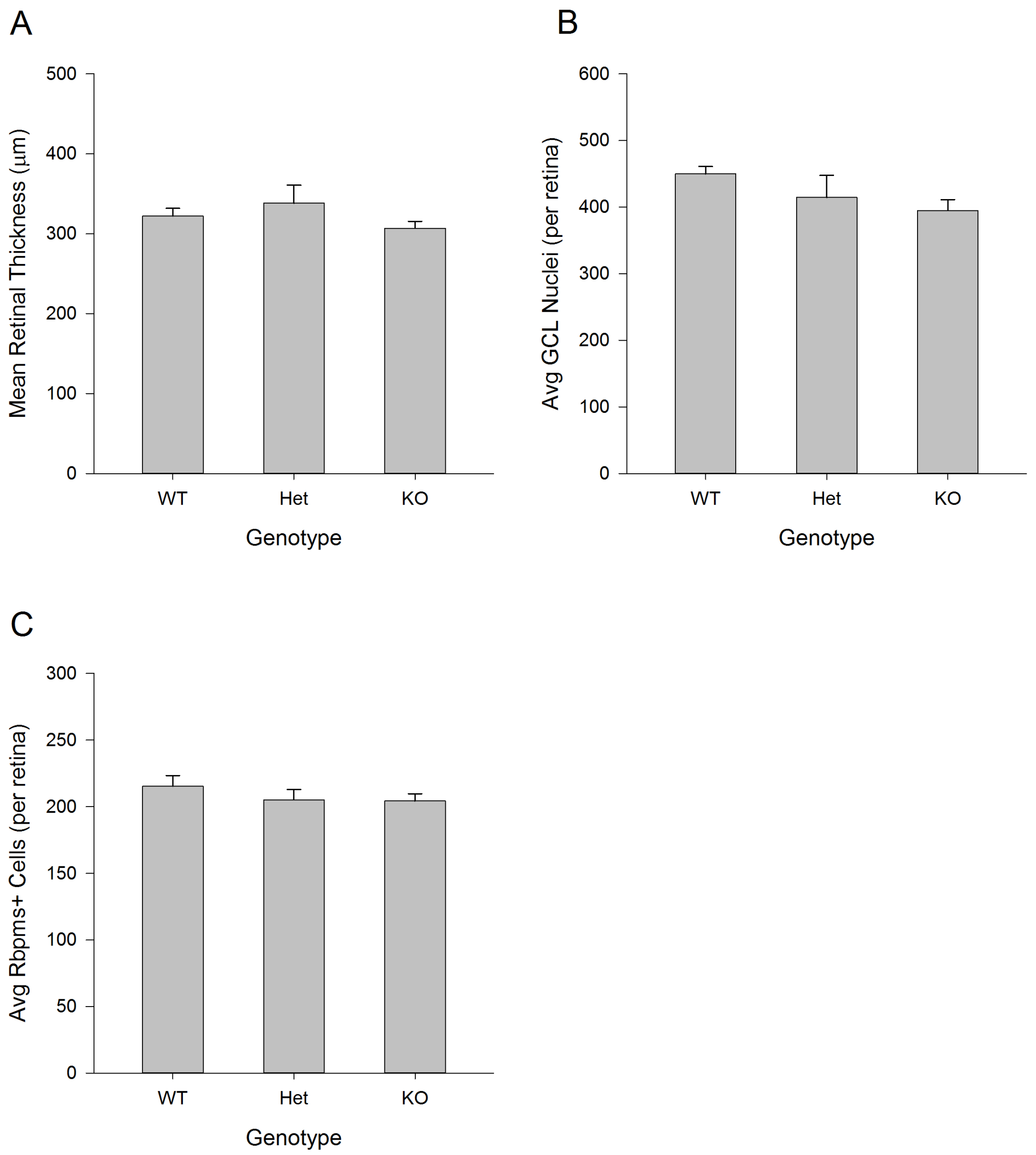

Supplemental Figure 1. *C1qa*-deficiency does not affect retinal morphology. Naïve retinas from *C1qa*$^{+/-}$ and *C1qa*$^{-/-}$ were compared to those of WT mice in all neuroprotective assays. **(A)** No statistical differences were observed between all three genotypes in total retinal thickness. Likewise, when total nuclei in the GCL **(B)** and Rbpms^{+} **(C)** cells in the retina were counted, no differences were determined. Therefore, quantifications indicating preservation of retinal thickness and neuron counts are not due to developmental differences within the retina between transgenic and WT mice. Mean ± SEM, n=5 per group.
